# Experience of General Practice Residents Caring for Dependent Elderly during the First COVID-19 Lockdown—A Qualitative Study

**DOI:** 10.3390/ijerph182312281

**Published:** 2021-11-23

**Authors:** Johann Augros, Frédéric Dutheil, Amanda C. Benson, Marie-Pierre Sauvant-Rochat, Gil Boudet, Catherine Laporte, Benoit Cambon, Guillaume T. Vallet

**Affiliations:** 1 Université Clermont Auvergne, CNRS, LaPSCo, CHU Clermont-Ferrand, WIttyFit, F-63000 Clermont-Ferrand, France; frederic.dutheil@uca.fr; 2 Department of Health and Biostatistics, Swinburne University of Technology, Hawthorn, VIC 3122, Australia; abenson@swin.edu.au; 3 Université Clermont Auvergne, Clermont Auvergne INP, CNRS, Institut Pascal, F-63000 Clermont-Ferrand, France; m-pierre.sauvant-rochat@uca.fr; 4 Université Clermont Auvergne, CNRS, LaPSCo, F-63000 Clermont-Ferrand, France; gil.boudet@uca.fr (G.B.); guillaume.vallet@uca.fr (G.T.V.); 5 Université Clermont Auvergne, Clermont Auvergne INP, CHU Clermont-Ferrand, CNRS, Institut Pascal, F-63000 Clermont-Ferrand, France; catherine.laporte2@uca.fr; 6 Université Clermont Auvergne, UPU ACCePPT, F-63000 Clermont-Ferrand, France; benoit.cambon@uca.fr

**Keywords:** occupational health, cognitive dissonance, resident, general practice, COVID-19, dependent elderly people

## Abstract

Background: Understanding the experiences of general practice (GP) residents caring for dependent elderly people during the first lockdown as part of the countries COVID-19 pandemic strategy. The aim was to explore themes that could explain the gap between the missions and values at the heart of GP practice during this period of strict isolation. Method: Qualitative study using an iterative approach. Semi-structured interviews were conducted with 13 GP residents using a pre-established interview guide. Audio recordings were transcribed verbatim. Data were analyzed according to a coding grid, developed using Nvivo software (NVivo Qualitative Data Analysis Software; QSR International Pty Ltd. Version Release 1.5.1 (940) 2021), to identify emerging themes. Results: Three themes emerged from this qualitative research: cognitive dissonance, psychosocial risks, and fear. General practice residents have lived in the paradox between care and deprivation of liberty of dependent elderly people. Conclusion: The results suggest that the GP residents experienced a form of work-related suffering in this situation of deprivation of liberty of dependent elderly people. The present research serves as a pilot study to explore how GP residents experienced their care of locked-up dependent elderly people.

## 1. Introduction

Moral distress is “psychological harm that occurs when people are forced to make or witness decisions or actions that contradict their fundamental moral values” [1]. The 2019 Coronavirus disease pandemic (COVID-19) has had an impact on health care workers around the world [2] and caused psychosocial risks [3].

Since the arrival of the COVID-19 pandemic and specifically during the first lockdown in France from 17 March to 11 May 2020, the freedom of every citizen was restricted, especially in social and medico-social establishments and services. The restrictions on liberty were much more restrictive for dependent elderly residents in institutional care than the general population [4]. Indeed, these people were forced to remain alone in their rooms. During this period, they were forbidden to come into physical contact with other residents or patients and were also isolated from their families. These exceptional individual health measures aimed at protecting the community were poorly tolerated by the dependent elderly people, leading to a resurgence of slippage syndrome [5]. This phenomenon refers to a “serious state of physical and psychological destabilization, including anorexia, malnutrition, withdrawal, and opposition”.

Residents in general practice (GP) are at the heart of care operations for dependent elderly people. During their professional training, they regularly take charge of dependent elderly people. They regularly work in a nursing home for older adults during their internships in private practice, or geriatric wards during their hospital internships. During the first lockdown, they took care of residents or patients who were in isolation as part of France’s COVID-19 strategy. They practiced medicine in a context of deprivation of freedom of dependent elderly people, resulting from a government decision. However, GP residents have a duty to respect a Code of Ethics [6] which states that all persons, their autonomy, and their will must be respected, without discrimination on the basis of their condition or beliefs. It is possible that working in these changed conditions could lead to psychological suffering such as moral distress for the GP residents [1] as caring for people who have been locked up against their will for health reasons can be difficult.

During residency, work-related suffering or exhaustion may arise [7]. As in every medical specialty, GP residents are exposed to psychosocial risks [8,9] and their training is demanding. They have a duty of care, which is necessary for quality patient care, and which stems from international recommendations that they must be familiar with [10]. They must be able to make decisions in the interest of the patients based on these recommendations and on the moral values they deem appropriate for each situation. Moreover, the investment in the field is tremendous, both in terms of time and emotion. Consequently, it seems particularly relevant to study the impact of COVID-19 from the perspective of the GP resident.

The objective of this study is to understand how the GP residents experienced this period of pandemic caring for dependent elderly people in government-mandated isolation. Their experiences in the context of their values as doctors [6] and the expectations imposed by the unprecedented context of this pandemic will be explored to understand the potential cognitive dissonance [11,12] that may influence both the clinical judgment of GP residents and their ability to adapt emotionally to deal with this new situation. We thus carried out a qualitative research study, based on individual interviews with GP residents who had been in contact with institutionalized or hospitalized dependent elderly people during the first COVID-19 isolation period.

## 2. Method

The present study follows a qualitative research design [13,14,15] to explore the experience of GP residents in dealing with dependent elderly people during the first COVID-19 isolation period. All GP residents in the Université Clermont Auvergne (UCA) who managed dependent elderly people during the first containment, related to the COVID-19 pandemic were invited to participate.

### 2.1. Sampling

Each GP resident (193 in total) received an email proposing participation in the study on 31 March 2021. With the exception of a few residents working exclusively in pediatrics during the first lockdown, the vast majority of GP residents have been in contact with dependent elderly people. There were three positive responses to this e-mail, and the first interviews took place shortly afterward. Subsequent interviews were arranged as a result of the snowball effect [16] from the initial participants recruited. Participants were asked at the end of the interview if they could suggest any residents who might be willing to participate in the study.

The interviews were conducted until the data were saturated following a reflexive approach [15,16]. In total, thirteen interviews were conducted.

### 2.2. Ethical Agreement

The Sud-Est VI personal protection committee judged that “this observational study, which is similar to an evaluation of professional practices, does not raise any particular ethical problem and does not fall within the scope of the regulations governing Research Involving the Human Person, within the meaning of Article L.1121-1 and Articles R.1121-1 and R.1121-2. According to this ethical agreement, we were able to carry out this research work after obtaining the oral non-objection of each participant.

### 2.3. Interview Guide

The interview guide (Appendix A) used for the semi-structured interviews was developed to study the possible cognitive dissonance [11,12] between, on the one hand, the values of the GP, guided *a priori* by the Code of Medical Ethics [6] and, on the other hand, the missions imposed by the government isolation restrictions during the first confinement period. The questions consist of open-ended questions grouped into four parts.

The “Opening” part includes an “ice-breaker” question, the purpose of which is to allow the participant to recall his or her internship, as well as to put the reflection into the context of the first lockdown.

Then, the second part asks the participant to define the values and missions of a GP in his or her own words; it also includes questions asking the participant to express his feeling of closeness to the elderly patients.

Then, the third part asks the participant to provide their point of view about the implementation of confinement measures and their impact on the dependent elderly people in particular; the aim is also to gather the lived experience of the possible consequences of the measures taken.

Finally, the fourth part aims to elicit the participants’ personal experiences observing the effects of the isolation on the dependent elderly people during the first lockdown. This last part takes into account what was said earlier, in particular the values and missions expressed at the beginning of the interview in relation to the specific situation of caring for dependent elderly people in isolation. Question 4.b is formulated in a very open way, to explore the idea of cognitive dissonance and identify any form of professional and personal suffering that the participant identifies.

### 2.4. Data Collection

The individual interviews were conducted by a researcher who was himself a GP resident. These interviews took place between 9 April and 23 June 2021. They took place either face-to-face, in a public or private place, in a setting conducive to confidential discussion, with complete freedom of expression; or remotely via the MSTeams software [17]. Each interview was recorded in its entirety in audio using a professional dictaphone, including those on MSTeams. All interviews (face to face + online) were recorded audio-only, the video option of the MSTeams software was not recorded to maintain consistency between interviews. This allowed for comparable transcription. The researcher followed the pre-established semi-structured interview guide (Appendix A). The audio recordings were transcribed verbatim, in French, into a text corpus by the researcher himself using Word software, version 2016 Microsoft 365 Apps for Enterprise. This was then translated into English after the analysis was complete. Confidentiality was ensured by using numbers instead of names (P01, P02, etc.) and by removing identifying information (e.g., all verbatims were transcribed in the masculine gender). All audio recordings and transcripts were saved on a password-protected computer.

The interviews were conducted until data saturation was reached [15,16]. This was decided by the researcher-investigator when the last interview did not provide any new emerging themes but provided ‘codable’ data in the coding grid that repeated ideas already discussed by the other participants.

### 2.5. Analysis

An iterative approach [18], i.e., mixed, both deductive and inductive, was developed to extract the emerging themes from the text corpus.

We first implemented a deductive approach [19], i.e., the development of a coding grid built on the basis of a hypothesis of a probable cognitive dissonance experienced by the GP residents during the first lockdown. This was an *a priori* theme identified in this type of situation [20].

Then we implemented an inductive approach [21], i.e., a back-and-forth between transcribed text and coding grid. We enriched the coding grid with elements identified in the interview text that were not covered by the grid produced *a priori*. Therefore, the coding grid evolved as the body of text was analyzed. It was constructed during the interviews, and the themes were established by repetition from the participants’ comments. It is the results that we discovered and not a predefined analysis that are in this coding grid, which is why it is in the Results section.

The context of expression was the adaptation of GP residents during the first lockdown, their point of view on the isolation of dependent elderly people. This was to target the psychosocial risks, particularly in relation to the cognitive dissonance that each participant may have encountered. Furthermore, through these different accounts of experiences, other psychosocial risk factors [22] emerged, as well as the fear felt during this period.

We used Nvivo [23] software to undertake the thematic analysis of the data from the interviews. This analysis was both manual and automatic thanks to the different functions of the software. The figures presented in this report were constructed using this software. The thematic analysis was carried out as the interviews were conducted. After each transcription, we had word clouds made with the Nvivo software. The values of general practice and the tasks imposed by containment emerged as well as cognitive dissonance. We then noticed during the interviews that psychosocial risks were expressed by all participants. The same was true for fear.

### 2.6. Check

The interview guide and coding grid were developed and validated by two other researchers in the field.

In addition, a check of the qualitative methodology, in particular the factors that could influence the interviews, was carried out using the COREQ checklist [24] during each stage of the construction of this study. This check is detailed in the Discussion section, sub-section Limits.

## 3. Results

A total of thirteen interviews were conducted, with no refusals or dropouts. We constructed a model for understanding the experiences of the thirteen participants (Table 1), all GP residents, who managed dependent elderly people during the first COVID-19 pandemic containment.

### 3.1. Coding Grid

**Figure 1:**
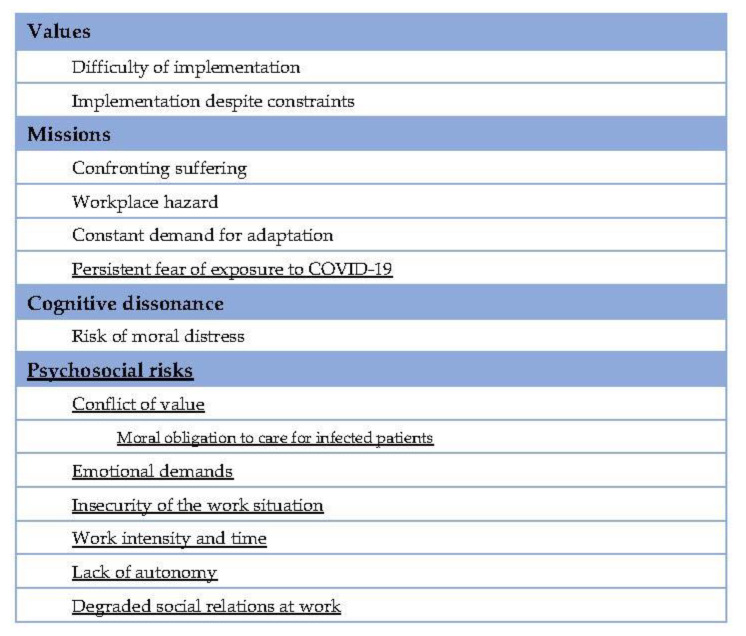
Coding grid. Underlined: themes or sub-themes that emerged from the inductive approach.

The coding grid (Figure 1) was developed based on the hypothesis of a probable cognitive dissonance experienced by the participants between their values as a GP, guided *a priori* by the Code of Medical Ethics [6], and their new requirements during the first COVID-19 lockdown period, in particular, their role caring for isolated dependent elderly people.

According to the scientific literature [12,20,25], we had reasons to believe that living and working in cognitive dissonance leads to a form of moral distress. We built part of our coding grid on the hypothesis that the GP residents had difficulties and felt a form of suffering when they associated their work values with the reality of the requirements imposed by the lockdown, the isolation of the dependent elderly people.

During the interviews, we noted that the participants spontaneously mentioned their difficulties in dealing with dependent elderly people, particularly when these people were isolated. The theme of psychosocial risks emerged as a code (or node, depending on terminology) in the coding grid. The theme of fear felt during this period also emerged consistently among participants and constitutes a sub-code of the “Missions” code. The themes that emerged from the inductive approach [21] are underlined in the coding grid (Figure 1).

Principle of the coding grid (Figure 1): the content of each verbatim interview, in terms of sentences or paragraphs, was coded according to one of the codes or sub-codes present in the coding grid.

### 3.2. Values and Missions

The values and missions of the GP most frequently mentioned by the participants corresponded to the vision of the participating residents. These future GPs defined their role as listening, professionalism, respect, and availability as the values that the participants agree on. The missions of support, follow-up of all pathologies, and prevention were mentioned by most participants.

### 3.3. Cognitive Dissonance

The isolation constraint faced by the dependent elderly people during the first confinement period caused difficulties for the participants (GP residents) to implement the values and missions they identified as being important leading to a contradiction or cognitive dissonance. The following paragraphs outline the participants’ expressions of the cognitive dissonance they experienced. The verbatim quotes are in inverted commas.

For Participant 1 (P01), there seems to be a dissonance between the need to isolate dependent elderly people and the associated consequences, “*Completely closing down* *dependent elderly people, from a health point of view, actually there was… Sigh. It had to be done. But I think that it was really very, very deleterious. On the psychological side of the elderly*”. This idea is shared by P03,

“*in my opinion, it was an important measure because the nursing homes were indeed a bit overwhelmed by the epidemic. Well, it affected the elderly a lot, but I think it was still very hard for the residents to live with. Not seeing their families. Some people in the nursing home are at the end of their lives, um… I think they had a very hard time living with the fact that they couldn’t have any contact with their family, or visual contact, or direct contact. That’s it…*”

Several participants highlighted their disagreement or resentment with this isolation, such as P02,

“*You see, I didn’t take it well, for example, I thought that, in fact, they were robots who… well, I thought it was so bad of the administration not to authorize the families to come, or else only at the end, at the end of the line*”

and P04:

“*It hurt my heart… Personally, it hurt my heart to think that, at that age… perhaps also for those who don’t have cognitive problems or not very many, they are already aware that they are coming to the end of their life. And that they may still have things to live for, but not much. And now we are depriving them of that.*”

Some participants expressed their assessment of the benefit/risk ratio concerning the isolation of dependent elderly people such as P05*,*

“*but on the other hand, you think, an elderly person who won’t be around for much longer, who doesn’t see his family, who is actually dying of loneliness. And… isn’t it perhaps better to leave with the COVID than to die of loneliness alone in their room? I mean, I think it’s… it’s hard*”

or P06:

“*having a COVID and at least you’ve seen your children, your grandchildren. I don’t know… because when you’re in a nursing home you’re 90 years old, your days are numbered, I think, it’s perhaps more important to see your family than to say to yourself “well at least I didn’t get the COVID but…” well that’s it. That’s my opinion.*”

Several expressed their pain, such as P07, “*our mission as careers is somewhat undermined in these situations*”, and P12: “*I, who advocate humanity, it was distressing that patients could no longer see their families”.*

P11 describes the discrepancy between the missions imposed by isolation and what the dependent elderly people could expect, “*I had the impression that I was spending quite a lot of time trying to sell… well, to apologize, we’ll say, for the situation to the patients because they were locked up.* P13 also expressed regret at having been involved in this care: “*I thought it wasn’t suitable, that it was too hard for them, well… silence… that we could have done things differently. And should have done differently”.*

**Figure 2:**
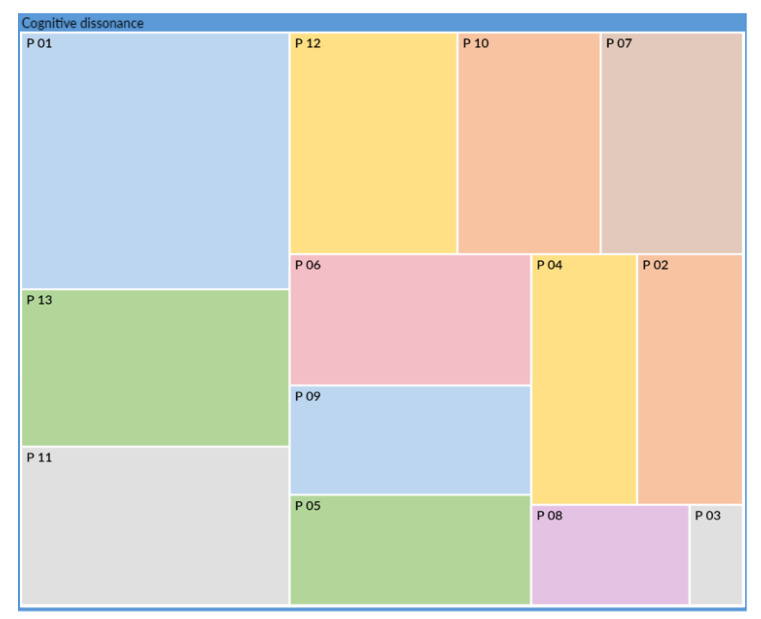
Proportion of evocation of a form of cognitive dissonance verbalized by each participant.

Figure 2 shows that cognitive dissonance was consistently evoked by participants when they recalled their handling of the dependent elderly people during the first lockdown. Each colored rectangle corresponds to sentences expressed by each participant, the area of each of these rectangles is proportional to the number of verbatim statements coded for each participant out of the total verbatim statements coded for cognitive dissonance. These are ranked in descending order of size. We can see that P01 (13 verbatims) expressed the most on cognitive dissonance, while P03 (2 verbatims) expressed little on this subject.

Participants described the contradiction between the need to isolate the dependent elderly people to protect them from the virus and the consequences that these precautionary measures could have on the lives of these people.

### 3.4. Psycho-Social Risks

Each participant mentioned at least one psychosocial risks factor. Real suffering was expressed by the participants concerning their experience of dealing with isolated dependent elderly people during the first lockdown. The following verbatim statements were coded (Figure 1) according to the psychosocial risks factors defined by the Copenhagen Psychosocial Questionnaire (COPSOQ) [22]. The percentages indicated correspond to the weight of each sub-code in relation to the others, within the psychosocial risks code (Figure 1).

Conflict of value (32.6%)

P13 explains how he experienced being forced to isolate the dependent elderly people, “*For me, it’s true that the patients wanted to see their relatives. For many, it’s their only reason to survive and live. And it’s true that, well, to take that away from them… it seemed rather contrary to my vision of things.*” Other participants emphasized the heartbreak of working in these conditions, such as P03: “*we found it difficult not to be able to… spend the last moments they have left… not to be able to spend them with their family. So that’s difficult to live with psychologically, as a career*”, P07: “*for me it was difficult to accept… to know that they were cut off from their social link, which for them was more important than their health problem*” and P09: “*at the time when we closed access to all the families, that’s when it was hardest*”.

P11 explained the embarrassment he felt in defending an idea that was in opposition to his vision and values: “*it’s difficult to explain to people that, in fact, we are putting them in their own corner to make them healthy*”.

Emotional demands (19.4%)

This psychosocial risk factor is more general. When working with sick people, there is a confrontation with their physical, psychological, and social suffering. However, some specificities related to the isolation of dependent elderly people emerged, as expressed by P01, “*I found myself faced with people who, um… slipping syndromes, depressive syndromes in the process of starting, etc.*”, P07: “*it was difficult to accompany patients who cry, who are sad, who let themselves slip…*” and P13*: “many patients who were finally able to verbalize. Even the most demented ones, in fact, ‘if I don’t see my family there’s no point in me continuing to live’, so it was still quite hard for them […] we felt a real malaise for the patients.*”

P04 gives the example of a patient in a nursing home whose situation disturbed him, “*For him it was horrible… and even for his family who would have liked to be there to see him, to help him, to support him… and so it **was** difficult to accept the ban on visits”.*

Insecurity of the work situation (6.3%)

During this semester, some of the participating GP residents were confronted with an imposition of tasks concerning the management of patients with COVID-19. P10, “*I don’t remember if we were asked, well… I don’t remember if we were asked if we agreed to go*”. This is an “uncontrolled change in the role and working conditions” as defined by the INRS(21). Conversely, P12 was not allowed to take part in the care of patients with COVID-19, “*I wanted to take part in screening in the covidromes, etc., and I had two of my doctors who kindly told me that if I went to do that over there, I wouldn’t be coming back for 40 days and that they would quarantine me.*”

Work intensity and time (23.6%)

P01 reports his mental involvement due to the intensity of the work and having to work more hours in the management of the dependent elderly people, “*There was more investment”.*

P02 reports the long working days during this period, “*In the end, I was often the one doing the visits alone, even if I could always count on them* [the senior doctors]*… they were available. And um… they stayed late, I stayed late too.*”

Lack of autonomy (10.4%)

It was difficult for P06 to submit to a new regulation concerning the isolation of dependent elderly people and that the decisions were not their responsibility, “*Except that we were not informed. It was the management that took the decision.*” Other participants found themselves in a similar position, such as P09: “*We didn’t have much choice”.*

Poor social relations at work (7.6%)

P02 emphasized the deterioration in relations with the paramedics, “*it was a bit complicated, there were nurses who were a bit demanding in terms of protection and all that… “ha but we don’t have the right protection!*”

Other participants mentioned problems with the response from dependent elderly people, such as P12, “*When we went to see them, there was a lot more aggression, waiting, apprehension. Not at all as healthy a relationship as it could have been before the pandemic” […] “People were much more aggressive and the doctor-patient relationship suffered.*”

**Figure 3:**
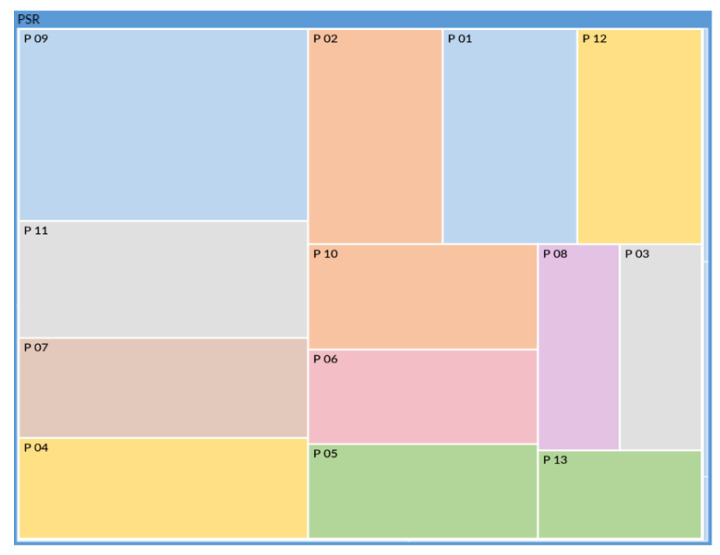
Proportion of statements of at least one psychosocial risks factor verbalized by each participant.

Following the same principle as for Figure 2, Figure 3 below shows that all participants expressed psychosocial risks factors concerning their experience of this situation.

Each colored rectangle corresponds to sentences expressed by each participant, and the area of each of these rectangles is proportional to the number of verbatims coded for each participant out of the total number of verbatims coded for psychosocial risks. These are ranked in descending order of size. We can see that P09 (23 verbatims) expressed the most psychosocial risks factors, and P13 (6 verbatims) expressed the least.

**Figure 4:**
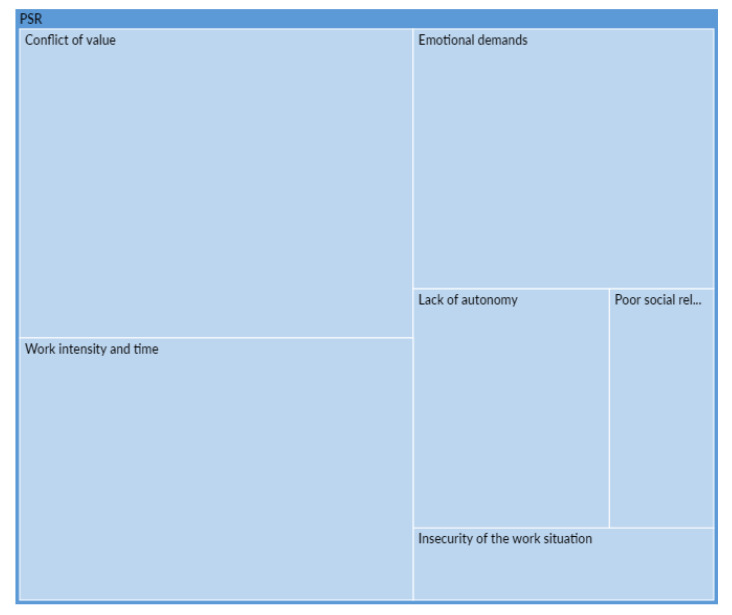
Proportion of psychosocial risks (PSR) factors mentioned by participants.

Figure 4 below shows the verbatim statements (144 in total) coded in each of the six psychosocial risks factors, i.e., the sub-codes of the psychosocial risks code (Figure 1). It shows that Value Conflict (32.6%) and Work Intensity and Time (23.6%) are the most represented psychosocial risks factors in the participants’ accounts of their experiences, together accounting for more than half of psychosocial risks factors.

Based on COPSOQ [22], the participating GP residents expressed their suffering in caring for their patients in these lockdown conditions. They were suffering seeing the dependent elderly people suffer, and their working conditions seemed to deteriorate during this confinement situation.

### 3.5. Fear

The fear felt during this experience was very present during the interviews. This is a sub-code of the Mission code, consisting of 31 verbatims. Only P03 did not mention fear when recounting his experience.

The persistent fear of exposure to COVID-19 was expressed through uncertainty by P02, “*At the first containment we didn’t really know what this virus was, it was apparently there, we had no way of testing*”, as well as by P06:

“*and then it’s true that we were worried because we didn’t know much about it, we didn’t know if there were other modes of transmission that we didn’t know about so um… probably that the care was less; maybe less good than if we weren’t stressed about it what. “*

P10 confided, “*I was falsely reassured”.*

The fear expressed by the GP residents during the interviews is the fear of catching COVID-19 themselves. This is an awareness of the danger they were exposed to during this period while working.

## 4. Discussion

This study was conducted to explore how GP residents experienced their practice during the first, and more serious, lockdown as a response to the pandemic, particularly in their role in caring for the elderly.

We have reported how the GP residents experienced the management of the dependent elderly people during the first lockdown related to COVID-19. The idea was to explore a possible cognitive dissonance between their values of medicine and the tasks imposed by the first lockdown (missions), notably the isolation of the dependent elderly people. The results suggest that working in this situation of cognitive dissonance is at the origin of psychosocial risks factors and a feeling of fear, and a source of suffering such as moral distress that these GP residents experienced. Three themes, therefore, emerged from this qualitative research: cognitive dissonance, psychosocial risks, and fear.

This research work is in line with the studies referenced below, concerning the widespread and universal suffering of caregivers during the COVID-19 crisis. Indeed, caregivers, including residents, have experienced a trying time with this pandemic. Suffering was already present, and it would seem that these working conditions have amplified this suffering.

### 4.1. Cognitive Dissonance

The initial aim was to see whether the GP residents had experienced the contradiction of cognitive dissonance. This theme was explored through a deductive approach [19]. That is, the interview guide and coding grid were designed to bring out this theme. Cognitive dissonance was raised by each study participant, particularly the need to isolate dependent elderly people and the associated consequences of this strategy. Participants also reported disagreement or resentment with this isolation. In addition, they reported their assessment of the benefit/risk ratio of isolating dependent elderly people and expressed a contradiction with their professional values. The various statements made during the interviews showed a discrepancy between the tasks imposed by the isolation and what the dependent elderly people were able to expect. The participants found themselves in a psychologically uncomfortable state as described in the study by Sweeney, J. et al. [26]. This study also provides a scale for measuring cognitive dissonance which may be of interest for further studies on this topic.

### 4.2. Psychosocial Risk

Psychosocial risks themes, like fear, emerged following an inductive approach [21]. It was decided to classify the psychosocial risks according to the COPSOQ risk factors [22]. We found that value conflict was the psychosocial risk factor most expressed by the participants. This seems quite logical because this sub-theme has similarities with cognitive dissonance. The percentages given in the psychosocial risks sub-section of the Results section are for illustrative purposes only and are not statistically representative. As the interview guide was designed to assess the possible emergence of cognitive dissonance, it is not surprising to find a strong expression of value conflict among the participants. This sub-theme implies an alteration of moral awareness related to the situation experienced. This is an additional argument for evoking a form of moral distress. Other participants frequently expressed psychosocial risks factors were work intensity, time, and emotional demands. These results suggest that the GP residents were highly engaged in their work during the period of the first lockdown. They suggest that they spent many hours in contact with the dependent elderly people and were directly confronted with the suffering of these isolated patients.

The results are consistent with data in the literature concerning unhappiness [27], burnout syndrome [7,28,29], or suicide [30,31] among residents of all specialties, including GP residents, in different regions of the world. Even when not directly asked about work-related suffering, it emerged consistently among the participants. The results suggest that suffering is present among GP residents in this work environment.

### 4.3. Fear

Almost all the participants expressed their fear of the situation they were in. This emotion seems legitimate when working in a context of uncertainty that has a potential danger to oneself. The participating residents came across people with COVID-19 during their residency in the first lockdown. They were then exposed to this new disease, which could have serious consequences for their own health, and probably felt insecure. This was also the case in other parts of the world such as China [32].

Working in fear increases anxiety [33]. Fear related to the COVID-19 pandemic can have consequences on health [34]. Our study suggests that the GP residents felt this fear. This factor should be taken into account given the vulnerability of this population [35], particularly with regard to the risk of suicide [36]. and perhaps be considered in how residents are prepared in the future prior to embarking on this type of internship.

### 4.4. Perspectives

In addition to the suffering that may eventually put residents who have worked under the isolation conditions caring for dependent elderly people at risk of suicide, other problems may emerge from the findings of this study.

The fact that these future doctors are obliged to work in cognitive dissonance modifies their treatment of dependent elderly people. Indeed, to protect them from danger, they must confine them and therefore make them suffer. This paradox raises ethical questions. This imposed treatment of dependent elderly people raises questions about their place in society. The theme of ageism could be explored further in the future on the basis that this social class has been discriminated against under the pretext that the precautionary principle must be respected. Awareness of this problem may have caused additional suffering for the residents who worked in this situation.

It would be interesting in the future to explore how the residents are trained to deal with this type of difficulty in order to understand their current fragility in coping and adapting to situations similar to the lockdown related to the COVID-19 pandemic.

It would be relevant to draw parallels with the suffering experienced by people quarantined because of COVID-19 in Poland [37]. Furthermore, it might be appropriate to apply recognized methods to assess psychological stress in the most objective way possible [38]. This would allow the accumulation of comparable data on the adverse effects of lockdown worldwide.

### 4.5. Limitations

The limitations are presented in accordance with the COREQ checklist [24].

First, the conduct of the individual interviews. The investigator was the researcher who carried out this study. He is himself a GP resident. He is a man. He knew some of the participants, nine of the thirteen who participated. There may be a bias in the responses obtained because the participants and researchers knew each other before the study was carried out. However, the inclusion of participants was based on voluntary participation (all residents received the email invitation) and not by directly appealing to a network of acquaintances, in order to limit the Hawthorne effect [39]. A semi-structured interview schedule was also used to ensure a consistent approach with all participants. All participants knew that the researcher was one of their co-residents. The risk was that participants would see him as a peer rather than a researcher. To gain neutrality, the researcher explained at the beginning of each interview that they should try to see him as a researcher rather than as a resident, to avoid going outside the framework of the interview.

Concerning the target population, the results only represent those themes that were raised by residents with low- or medium-experience (1st and 2nd year). In the future, 3rd-year residents could also be considered.

The participants were aware that the researcher was carrying out work on the experiences of GP residents during the first lockdown, regarding the management of dependent elderly people. They did not know that one of the objectives was to assess their experience of cognitive dissonance associated with this situation. However, they did report this in their account of their experience. The interview guide was developed to encourage the expression of cognitive dissonance by starting from the values and missions of the GP and highlighting the adaptation the participant had to make during the first confinement. Several participants were unsettled by the first questions asking about values and missions. Nevertheless, this frontal and open approach allowed them to keep these notions in mind for the rest of the interview. It would appear that we have helped to bring out the cognitive dissonance by having participants focus on the notions of GP values and mission.

The transcripts of the interviews (face-to-face and online) were not returned to the participants. Only the first response intention was analyzable in this work. Any comments made by the participants afterward would have been influenced by the desire to evoke a form of cognitive dissonance. Indeed, the interview guide gradually reveals the response intentions sought by the investigator and was developed for this purpose. It was tested on two volunteers, not medical residents, beforehand. Reviewing it would influence and modify the responses and social desirability bias [40] may have been a factor.

The present research serves as a pilot study to explore how GP residents experienced their care of locked-up dependent elderly people. The iterative approach followed should have helped to reduce the bias associated with one person’s interpretation of the data.

## 5. Conclusions

This qualitative study identified three themes regarding the experiences of the GP residents who managed care for dependent elderly people during the first COVID-19 pandemic lockdown: cognitive dissonance, psychosocial risks, and fear. Managing isolated dependent elderly people in the context of a rapidly implemented and unprecedented precautionary principle was difficult for the participating residents. The results suggest that their decision-making may have been at odds with their moral values, and they may have experienced moral distress.

This research work has highlighted a form of psychological suffering among the GP residents. This suffering is quite general and is expressed both in the form of moral distress and in the form of other psychosocial risks. Workplace distress among interns in all specialties appears to be present in many parts of the world and existed prior to the arrival of the COVID-19 pandemic. It might be interesting to study the consequences of this situation on medical training, for example by drawing a parallel with the recent suicides of interns in France. A research project could study the state of the suicidal crisis among GP interns, and/or other specialties, and its evolution since the arrival of COVID-19.

It could also be interesting to study the consequences of the confinement of dependent elderly people for the purpose of collective prevention. This situation may give rise to ethical questions about the value placed on the dependent elderly people in our society.

## Figures and Tables

**Table 1:** Summary information about the thirteen participants and interviews.

**Participants’ Characteristics**	
Gender	Women: 8/Men: 5
GP resident progress in studies Age	2.5 ± 0.8 semesters 27 ± 0.7 years old
Place of work experience	-Geriatric medicine: 7 -Emergency or COVID unit: 3 -Other hospital specialties: 3
**Interviews characteristics**	
Duration	23 ± 5.5 min
Place	Face to face: 6/Online: 7

## Data Availability

The data supporting the reported results are stored on a computer with a security password. There is currently no publicly archived data.

## Appendix A. Interview Guide

1. Opening

a. Anonymous

b. Voluntary

c. No right or wrong answer, just the desire to collect opinions

d. Confidentiality, freedom of speech

e. “Ice-breaker: can you introduce me to your placement, which was carried out during the first lockdown?

2. GP values

a. What are the values/missions of a GP for you? (or even role?)

b. What are your values as a GP resident?

c. What do you think is the role of the GP in relation to the elderly and their medical care?

d. How do you see your professional future in medicine with regard to the aging of the population?

3. Pandemic COVID-19 and dependent elderly people

a. What is the place of general medical residents in the pandemic/pandemic management/patients…?

b. How do you judge the use of containment, especially for the former which has been the strictest so far?

c. How do you perceive the sanitary measures put in place in a nursing home during the first containment?

d. What do you think about the isolation of the elderly in the first lockdown?

e. How do you perceive the benefit-risk ratio of the isolation of dependent elderly people in relation to COVID? Other measures for dependent elderly people in a nursing home?

4. Likely cognitive dissonance and risk of moral distress

a. How do you judge the implementation of isolation of dependent elderly people in a nursing home in relation to the role/missions and values of a GP? Then in relation to your personal values?

b. How did you experience this isolation of dependent elderly people?

c. What was the impact of this isolation on your practice?

d. Would you have done anything differently if you had been a senior doctor? If so, what would you have done?
